# Association between Antipsychotics-Induced Restless Legs Syndrome and Tyrosine Hydroxylase Gene Polymorphism

**DOI:** 10.4306/pi.2009.6.3.211

**Published:** 2009-07-15

**Authors:** Chul-Hyun Cho, Seung-Gul Kang, Jung-Eun Choi, Young-Min Park, Heon-Jeong Lee, Leen Kim

**Affiliations:** 1 Department of Psychiatry, Korea University College of Medicine, Seoul, Korea.; 2 Division of Brain Korea 21 Biomedical Science, Korea University College of Medicine, Seoul, Korea.; 3 Department of Neuropsychiatry, Inje University College of Medicine, Ilsan Paik Hospital, Goyang, Korea.

**Keywords:** Restless legs syndrome, Antipsychotic, Schizophrenia, Tyrosine hydroxylase, Polymorphism

## Abstract

**Objective:**

Restless legs syndrome (RLS) has been reported to be more prevalent in schizophrenic patients who take antipsychotics. The cause of RLS is unknown but associated with dopaminergic deficiency. Tyrosine hydroxylase (TH) is the enzyme responsible for catalyzing the conversion of L-tyrosine to DOPA. The purpose of this study is to determine whether the TH gene Val81Met polymorphism is associated with antipsychotic-induced RLS.

**Methods:**

One hundred ninety Korean schizophrenic patients were evaluated by the diagnostic criteria of the International RLS Study Group (IRLSSG). The genotyping was performed by PCR-based methods.

**Results:**

Of the one hundred ninety schizophrenic patients, 44 (23.2%) were found to have RLS. Although there were no significant associations between TH genotypes or allele frequencies and RLS, when separate analyses were performed by sex (male or female), we detected significant differences in the frequencies of the genotype (χ^2^=6.15, p=0.046) and allele (χ^2^=4.67, p=0.031) of the TH gene Val81Met polymorphism between those with and without RLS in the female patients.

**Conclusion:**

These findings suggest that the TH gene Val81Met SNP might be associated with antipsychotic-induced RLS in female schizophrenic patients.

## Introduction

Restless legs syndrome (RLS) is characterized by an unpleasant sensation in, and the urge to move the legs.^1^ RLS is a common disease, but is often underdiagnosed, undiagnosed or misdiagnosed as other psychiatric, neurologic or musculoskeletal systemic disease.^2^,^3^ The estimated prevalence of RLS depends on ethnic samples or the design of studies, and varies widely from 1% to 15%.^4^,^5^ According to recent epidemiologic studies in Korea, the prevalence of RLS is 12.1% in Korean adults aged 40-69 years.^6^ Other epidemiological studies in Korea have shown that the prevalence of RLS is approximately 7.5%, and among those only 24.3% receive treatment.^7^

The cause of RLS is not yet clear, but a leading pathophysiologic theory involves dopaminergic deficiency.^8^ The fact that RLS patients show RLS symptom relief after taking levodopa^9^ or dopamine agonists^8^,^10^,^11^ is evidence for the theory that dopaminergic deficiency causes RLS. In addition, RLS symptoms are relieved quickly and nearly completely with low-dose medication, which indicates an improvement of symptoms due to activation of the dopamine system itself rather than from other secondary changes associated with activation of the dopamine system.^12^ Patients who take antipsychotics show RLS more frequently, supporting the RLS dopaminergic abnormality theory. Antipsychotic-induced RLS is known to be caused by blocking dopamine receptors.^13^

Tyrosine hydroxylase (TH) is the enzyme responsible for catalyzing the conversion of the amino acid L-tyrosine to dihydroxyphenylalanine (DOPA), and is a rate-limiting enzyme.^14^ TH is found in the cytoplasm of noradrenergic and dopaminergic neuronal cells in the locus coeruleus, ventral tegmental area, substantia nigra, adrenal medulla, and sympathetic ganglia.^15^ The TH gene is located in 11p15.5.^15^,^16^ Activation of TH reflects the increase of dopamine production. The most common mutation of TH is Val81Met polymorphism in exon 2, but it is not yet clear how Val81Met polymorphism changes TH activity.^17^,^18^ The difference of functional activity in TH caused by the Val81Met polymorphism is not known till today.^19^

Desautels et al.^20^ analyzed the Val81Met polymorphism using a sample of 92 patients with RLS and 182 controls. No significant difference was found. As TH plays a very important role in generating dopamine, many studies suggest the association of the Val81Met polymorphism and movement disorders, such as Parkinson's disease (PD) and RLS.^18^,^21^ The purpose of the present study was to determine whether Val81Met polymorphism is associated with antipsychotic-induced RLS.

## Methods

### Subjects

One hundred ninety unrelated Korean schizophrenia patients were enrolled at Korea University Hospital and collaborating hospitals. They were aged between 22 and 66 years (mean±SD: 39.6±9.2 years). All of the subjects were diagnosed with schizophrenia by experienced psychiatrists according to the Korean version of the Structured Clinical Interview for Diagnostic and Statistical Manual of Mental Disorders fourth edition, and had been treated with antipsychotics. All of the participants provided written informed consent to participate, and the study protocol was approved by the Ethics Committee of Korea University Hospital. Some findings from these subjects have been reported previously.^22^,^23^ Exclusion criteria were as followings: 1) the patients were too psychotic, agitated or mutistic to be inteviewed, 2) patients presented with other Axis I diagnoses, mental retardation, neurological disorder, head injury, history of alcohol or other substance abuse, and 3) patients had serious medical diseases or other conditions that could induce secondary RLS, such as severe anemia, renal failure, radiculopathy and peripheral neuropathy.

### Assessment of symptoms

We gathered data on each patient's sociodemographics, duration of illness, prescribed antipsychotics and chlorpromazine equivalent dosage. Every assessment was performed during the daytime (between 09:00 and 17:00 h). RLS was assessed using the International Restless Legs Syndrome Study Group (IRLSSG) diagnostic criteria and a paradigm of questions used in epidemiologic studies of RLS. All subjects were asked about the four essential diagnostic criteria of RLS; i) the urge to move the legs, ii) unpleasant sensations in the legs, iii) symptoms worsening during rest and relief by movement, and iv) symptoms worsening in the evening or at night. If the unpleasant sensations were caused by arthritis, neuropathy, sports injuries, vascular problems, positional discomfort, sleep starts, simple cramping, and psychotic agitation or tactile hallucinations, we did not consider them as symptoms of RLS. All four essential diagnostic criteria of RLS were required for patients who were diagnosed with RLS, and those that did not meet all four criteria were included in the non-RLS group. The severity of RLS symptoms was assessed using the IRLSSG rating scale (IRLS) for RLS.^24^

The evaluation of the degree of psychiatric symptoms was done using the Brief Psychiatric Rating Scale (BPRS),^25^ which is an 18-item observer scale designed to assess patients with major psychiatric disorders.

### Genotyping

Venous blood was drawn from each individual, and genomic DNA was isolated using Accuprep Genomic DNA Extraction Kit (Bioneer, Korea) according to standard procedures. Analysis was done using Polymerase Chain Reaction (PCR) and PCR-based Restriction fragment length polymorphism (PCR-RFLP). PCR amplification was performed with the forward primer 5'-ATC CCC TGC CTC TGT GTG CCA T-3' and the reverse primer 5'-TCA GGA ACT CAG CCC ACA CAG C-3', giving a 404 base pair (bp) product. The PCR product was digested by the restriction enzyme NIaIII for approximately 3 hours at 37℃, then the reaction mixture was analyzed by 2% agarose gel electrophoresis to ensure correct amplification of the DNA fragment. NIaIII cleaves the Met81 allele into three fragments of 173, 140, 91bp, and the Val81 allele into two fragments of 313 and 91bp.^26^

### Statistical analyses

We performed the Hardy-Weinberg equilibrium test to assess the goodness of fit of the data. We analyzed the categorical data using the chi-square test, and evaluated the differences between continuous variables to establish whether there was an association between RLS and genotypes by using Student's t-test or one-way analysis of variance. Statistical analyses were carried out using Statistical Package for Social Sciences (SPSS) for Windows (SPSS Inc., Chicago, IL). All statistical analyses were two-tailed, and the level of statistical significance was set at p<0.05.

## Results

Among the 190 schizophrenia patients of our sample, 44 (23.2%) patients were diagnosed as having RLS, and 146 (76.8%) were included in the non-RLS group based on the IRLSSG diagnostic criteria. The sex, age, height, weight, duration of illness, duration of medication, treatment, chlorpromazine equivalents, proportion of typical (or atypical) antipsychotics did not differ significantly between the RLS and the non-RLS (Table 1). However, IRLS score and BPRS score were subsequently significantly higher in the RLS than in the non-RLS (t=9.746, p<0.001; t=2.266, p=0.025) (Table 1).

The genotype frequencies did not deviate from Hardy-Weinberg equilibrium. There were no significant differences in the genotype, allele and allele carrier frequencies among the RLS and the non-RLS groups. However, when-separate analyses were performed by sex (male or female), we detected significant differences in the frequencies of the genotype (χ^2^=6.15, p=0.046) and allele (χ^2^=4.67, p=0.031) of TH gene Val81Met polymorphism between RLS and nonRLS only in the female subjects. There were no significant differences in the genotype, allele, and Met allele carrier frequencies between RLS and non-RLS in male subjects (Table 2).

## Discussion

In this study, we investigated the association between antipsychotic-induced RLS and the TH gene Val81Met polymorphism, among schizophrenia patients who took antipsychotics. There were no significant differences in the genotype, allele and Met allele carrier frequencies between RLS and non-RLS groups. However, when separate analyses were performed by sex (male or female), there were significant differences in gene variants between RLS and non-RLS only in female subjects.

Epidemiological studies of RLS have previously reported higher prevalence in women than men. Berger et al.^27^ reported the overall prevalence of RLS was 10.6%, and women were twice as likely than men to be affected in the German population. Hogl et al.^28^ conducted a community-based study of prevalence of RLS, and the prevalence of RLS was 10.6% and higher in women (14.2% in women, 6.6% in men) in the Austrian population as well.

Kritzer et al.^29^ suggested estrogen and progesterone appear to be potent regulators of the catecholamine innervation of the primate prefrontal cortex. Such regulation is anticipated in the sex difference observed in prefrontocortical development and function. McDermott et al.^30^ directly compared striatal dopamine metabolism in gonadectomized male and female CD-1 mice treated with estrogen or oil vehicle. Estrogen-treated female mice showed increased dopamine release compared to oil-treated females. Estrogen did not affect striatal dopamine concentration or release in males. These results suggest that under conditions of equal hormonal status, striatal dopamine turnover and concentrations are differentially affected by sex. The results also suggest that estrogen can exert substantial effects on striatal dopamine metabolism by acting specifically in females to increase neuronal dopamine synthesis and release.

Some researches reported that antiestrogens (AEs) and estradiol (E) change dopamine turnover rates.^31^ In addition, Desautels et al.^32^ suggested that the high activity allele of the monoamine oxidase-A (MAO-A) gene may be involved in modifying the severity of RLS manifestations in females.

TH plays an important role in the synthesis of dopamine. Furthermore, in the previous study, subchronic antipsychotic medication reduced TH immunostaining in the striatum and caused shrinkage of dopaminergic cell bodies in substantia nigra.^33^ Lerner et al.^34^ reported changes of TH Vmax (maximal transport capacity) after chronic haloperidol administration and suggested that TH protein expression is elevated by long-term haloperidol treatment. Therefore, we hypothesized TH to be associated with antipsychotic-induced RLS in schizophrenia patients.

Because the symptoms of RLS are similar to those of akathisia, the differentiation is important. Although RLS and akathisia can overlap in some patients,^22^ we tried our best to differentiate between them based on the facts that symptoms of akathisia include the inner feeling of restlessness, it is not restricted to the legs, and is a daytime phenomenon.

This study has several limitations. First, we did not check serum iron levels. Because iron is the main cofactor of TH's catalysis,^14^ it is possible that our results reflect differential levels of serum iron. Second, our subjects were taking a various antipsychotics which have different mechanisms of action. However, chlorpromazine equivalents, and a proportion of typical (or atypical) antipsychotics did not differ significantly among the RLS and the non- RLS groups. Therefore, taking a broad spectrum of antipsychotics should not affect significantly the results. Third, we cannot exclude presence of population stratification bias. However, we do not think that stratification bias should be considered seriously in our sample because the Korean population is characterized by a genetic homogeneity.^35^ Fourth, the relatively small sample size limits the generalization of our findings. Because our sample size is relatively small, our data cannot exclude the possibility that TH Val81Met polymorphism has an influence on susceptibility to antipsychotic-induced RLS.

Taken these limitations together, further investigations involving additional genes and markers and larger samples are warranted to fully understand the genetic pathophysiology of RLS.

## Figures and Tables

**TABLE 1:**
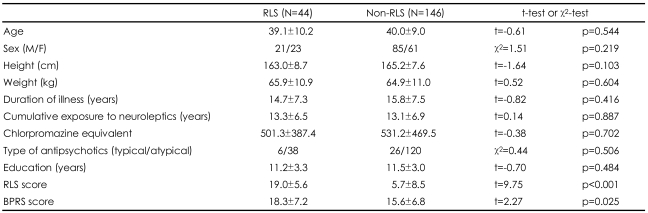
Differences in the demographics and clinical characteristics between RLS group and non-RLS group in schizophrenia

The values are mean±standard deviation (SD). RLS: restless legs syndrome, BPRS: brief psychiatric rating scale

**TABLE 2:**
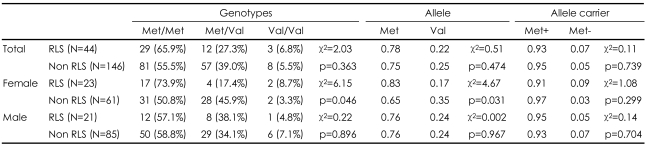
Genotypes, allele, and allele carrier frequencies of Val81Met polymorphism of the tyrosine hydroxylase gene among schizophrenic patients with and without RLS

RLS: restless legs syndrome, N: number of subjects
